# Teaching the *Rational Use of Medicines* to medical students: a qualitative research

**DOI:** 10.1186/1472-6920-12-56

**Published:** 2012-07-19

**Authors:** Karina Pavão Patrício, Nycholas Adriano Borges Alves, Nadja Guazzi Arenales, Thais Thomaz Queluz

**Affiliations:** 1 Departament of Public Health, Univ. Estadual Paulista - UNESP, Botucatu School of Medicine, Botucatu, SP, Brazil; 2 Department of Internal Medicine, Univ. Estadual Paulista - UNESP, Botucatu School of Medicine, Botucatu, SP, 18618-970, Brazil

**Keywords:** Rational Use of Medicines, Education, Medical, Undergraduate, Prescribing, Prescription, Drug

## Abstract

**Background:**

Prescribing is a complex and challenging task that must be part of a logical deductive process based on accurate and objective information and not an automated action, without critical thinking or a response to commercial pressure. The objectives of this study were 1) develop and implement a discipline based on the WHO’s Guide to Good Prescribing; 2) evaluate the course acceptance by students; 3) assess the impact that the Rational Use of Medicines (RUM) knowledge had on the students habits of prescribing medication in the University Hospital.

**Methods:**

In 2003, the RUM principal, based in the WHO's Guide to Good Prescribing, was included in the official curriculum of the Botucatu School of Medicine, Brazil, to be taught over a total of 24 hours to students in the 4th year. We analyzed the students' feedback forms about content and teaching methodology filled out immediately after the end of the discipline from 2003 to 2010. In 2010, the use of RUM by past students in their medical practice was assessed through a qualitative approach by a questionnaire with closed-ended rank scaling questions distributed at random and a single semistructured interview for content analysis.

**Results:**

The discipline teaches future prescribers to use a logical deductive process, based on accurate and objective information, to adopt strict criteria (efficacy, safety, convenience and cost) on selecting drugs and to write a complete prescription. At the end of it, most students considered the discipline very good due to the opportunity to reflect on different actions involved in the prescribing process and liked the teaching methodology. However, former students report that although they are aware of the RUM concepts they cannot regularly use this knowledge in their daily practice because they are not stimulated or even allowed to do so by neither older residents nor senior medical staff.

**Conclusions:**

This discipline is useful to teach RUM to medical students who become aware of the importance of this subject, but the assimilation of the RUM principles in the institution seems to be a long-term process which requires the involvement of a greater number of the academic members.

## Background

Prescribing is a complex and challenging task which must be based on accurate and objective information and not an automated action, without critical thinking or a response to commercial pressure. There are worldwide evidences of poor prescribing due to errors, polypharmacy, and inappropriate or irrational prescribing [1]. When medicines are prescribed or used erroneously, they pose serious health risks to the patient and significant associated economic implications [2,3].

Factors responsible for poor prescribing have been identified, such as deficiency of training, failure to perceive the importance of the task, lack of identifying the errors, and increasingly therapeutic options [4-6]. Reports from medical students show they do not feel prepared to prescribe [7,8]. First-year doctors are neither confident nor competent in writing a prescription corroborating the lack of undergraduate and postgraduate education in prescribing [9-12].

To overcome these difficulties, the World Health Organization produced the Guide to Good Prescribing [13] which takes the medical student through a structured problem-solved six-step process in choosing and prescribing a suitable drug for an individual patient. The WHO's Guide is based on the concept of *Rational Use of Medicines* (RUM) which requires patients to receive appropriate medications for their clinical needs, in proper individual doses for the correct period of time at a low cost for them and the community [2,3,14,15].

In prescribing a treatment, the doctor can choose between drug therapy, a combination of drug and non-drug therapy or only a non-drug approach. In the case of a drug based therapy using RUM is essential since it is a process that involves decisions made based on the efficacy, safety, convenience and cost. Furthermore, the correct prescription with the guarantee of access to the prescribed medication and adequate dispensing followed by the proper use by the patient is also part of the RUM principal. [3].

Considering the deficiency showed by young doctors in prescribing efficiently, in 2003 the RUM teaching was included in the official curriculum of the Botucatu School of Medicine, Brazil, as a mandatory discipline taught over a total of 24 hours, during one semester of the academic year. This discipline, based on the WHO's Good Prescribing Guide [13], trains students to learn a logical deductive process for selecting medicines according to the RUM principles (i.e., efficacy, safety, convenience and cost) and to write a correct prescription.

Based on these facts, the objectives of this study were 1) develop and implement a discipline based on the WHO’s Guide to Good Prescribing; 2) evaluate the course acceptance by former students; 3) assess the impact that the RUM knowledge had on the students prescribing in the University Hospital environment.

## Methods

*Site*: The Botucatu School of Medicine, São Paulo State University (UNESP), is a 49-year old public institution located in the city of Botucatu, State of São Paulo, Brazil. It has a medical curriculum of six years, with 90 students per year. It uses a traditional teaching methodology and, in a small scale, problem-based learning. The curriculum includes two years of basic sciences, two years of clinical medicine and two years of internship.

### Development and description of the discipline

The subject RUM was included in the official curriculum of the Institution in 2003 and is taught for the 4^th^ year of medical school over a total of 24 hours, during one semester of the academic year.

Objectives of the discipline: it is expected that by the end of the course students obtain the following competences:

1. Adopt a critical attitude regarding the search, selection and analysis of the different medicine information sources.

2. Adopt individualized criteria to choose adequate drugs for every clinical condition according to the RUM principles (efficacy, safety, convenience and cost).

3. Adopt criteria to indicate individualized treatment (drug and/or non-drug therapy) for every patient.

4. Write complete and accurate prescriptions.

5. Provide patients with information, instructions and warnings regarding the prescribed therapy.

6. Check all of the steps used to solve the problem, preparing a therapeutic plan in agreement with the patient.

**Figure 1:**
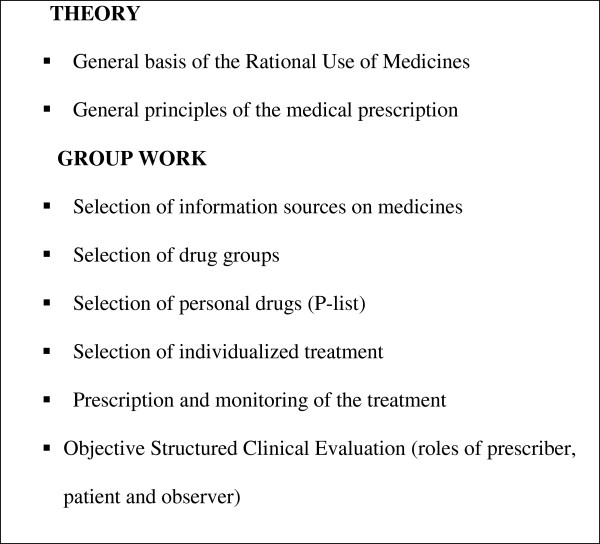
Theory and practical content of the discipline.

Organization: the discipline includes two theoretical classes for the whole group, five modules for group activity, one written cognitive assessment and another exam on abilities and competences by means of the Objective Structured Clinical Evaluation (OSCE), as shown in figure 1.

Teaching methodology: for tutorial activities, where the problem-based learning technique is used [16], each group of 10 students has a facilitator previously trained in the teaching of RUM according to the Teacher’s Guide to Good Prescribing [17]. The discipline is given throughout a week, enabling more concentration in the proposed activities, similarly to an immersion method.

The problem-based approach first critically discusses the information sources on medicines. Then, clinical problems lead students to develop a personal drugs (P-drugs) list for a specific disease. The P-drugs list is an evidence-based drug selection according to criteria (i.e., efficacy, safety, convenience and cost) determined in advance by each physician [18]. It contains the medicines which were chosen to be prescribed regularly. For this, students must review the pathophysiology of the disease, identify the pharmacological intervention sites, select potentially useful pharmacological groups, know the pharmacodynamic and pharmacokinetic properties of the groups as well as the respective medical evidences.

Subsequently, problems with patients presenting different comorbidities, those receiving other medications and/or with inappropriate life habits are introduced and the students must choose an individualized treatment for each one of them. Students must specify the therapeutic objectives, choose the therapeutic strategies and look for the drugs that might meet the individual needs of the patients in the previously prepared P-drugs list, testing if the list meets the needs of all of the patients.

Finally, the students write the prescription for these patients, with legible handwriting, clearly and without abbreviations, providing also the complete instructions for the administration and the warnings. In addition, students are instructed to include non-pharmacological actions for each case and encouraged to always ask if patients have any doubts and to check if they understood the prescription and the instructions provided.

The final assessment is performed by a written test with multiple choice questions and by a practical test using the Objective Structured Clinical Evaluation technique, where each student goes by three different stations, having the role of the physician, patient and observer.

### Assessment of the discipline by the students

A Student Assessment Form was developed with constituent questions (wh-questions), regarding to both teaching methodology and content of the discipline. These forms were given to students, whether or not on an anonymous basis, even if they were not filled out. The most significant and/or frequent responses to constituent questions were reported.

### Appraisal of this type of knowledge by former students of the discipline

In 2010, the impact of the discipline on the medical practice of former students was verified by qualitative methods [19-21]. A questionnaire with 17 closed-ended rank scaling questions was formulated and distributed at random to past students of the discipline, i.e., Interns and Medical Residents who still worked in our hospital. The former students were identified through their academic registers. Two hundred questionnaires, corresponding to 50% of the past students who still belonged to the institution at the time of the research, were delivered directly by hands. The responses should be returned on an anonymous basis to the professor's office.

In addition, a homogenous sampling of 25 responders who met the criterion of highest grades in the discipline were invited for a single semistructured interview approaching the following aspects: 1) Were there changes in your behavior on medicines selection and good prescribing practices? 2) How has the applicability of RUM in the medical practice of the institution been accepted or used? 3) Do you have any suggestion for improving the discipline? 4) Do you have any suggestion for introducing and disseminating the RUM in the Institution? This interview was audiotaped and later transcribed so as to provide the word-for-word text needed for the content analysis. The sample was closed utilizing the criterion of theoretical saturation [19-21], i.e., it was considered that the incorporation of additional interviews would make little significant contribution regarding to the objectives initially considered for this study.

The research was approved by the Research Ethical Committee of the Botucatu Medical School (no. 520/08).

## Results

### Assessment of the discipline by the students

From 2003 to 2010 the discipline was given to 720 students and in a scale from 0 to 10, in which 0 means minimum and 10 maximum, the mean grade given by students to the discipline was 8.9 ± 0.3 and to the tutor 9.1 ± 0.2.

In constituent questions, the most frequent responses were on the importance of proper drug prescription, stressing the fact that they had not yet thought about this subject. Students considered the teaching methodology and assessment adequate, and enjoyed the contact with tutors. However, they regretted the fact that in other disciplines they had taken, the choice of medicines is not based on the logical thinking used in the discipline, and rather, they use a pre-established “recipe” offered by the responsible people.

### Appraisal of the RUM knowledge by former students of the discipline

From the 200 questionnaires distributed, 75 (37.5%) returned: 58.6% from students of 5^th^ (S5) and 6^th^ (S6) years and 41.4% from Medical Residents (MR 1 to 4) (Table 1). The majority of the responders considered the knowledge of RUM very important for their medical practice (38.7%, always and 48%, sometimes). However, 37% reported that RUM is rarely remembered or demanded in other disciplines. In fact, frequently they are not stimulated to use this practice by both older residents and senior medical staff.

**Table 1:** Percentage of responses (n = 75) to the closed-ended rank scaling questions in the appraisal of the RUM knowledge by former students of the discipline

**Statement**	**A (%)**	**ST (%)**	**R (%)**	**N (%)**	**IDR(%)**
The knowledge obtained on RUM was important to my medical practice	38.7	48	9.3	0	4
The content of the discipline was remembered in both other disciplines and discussion groups.	4	44	37.3	1.3	13.3
I have been stimulated by professors and medical residents to practice the RUM in my daily academic activities	18.7	34.7	40	6.7	0
When I select medicines I follow the RUM principles (efficacy, safety, convenience and cost)	54.7	32	6.7	5.3	1.3
I seek information on medicines in non-commercial sources (text-books, scientific articles, non-commercial sites, etc.)	38.7	37.3	18.7	5.3	0
Teachers have been my main source of information on medicines	16	69.3	9.3	2.7	2.7
When I make a prescription I am stimulated by professors and residents to use the generic name of the medicines	44	26.7	20	6.7	2.7
When I make a prescription I am stimulated by professors and medical residents to avoid abbreviations	17.3	20	41.3	20	1.3
My prescriptions have a legible handwriting	88	9.3	0	1.3	1.3
I have been stimulated to know the side effects and interactions of the medicines I prescribe	13.3	50.7	28	6.7	1.3
I have been stimulated to completely know the posology of the medicines I prescribe	14.7	33.3	33.3	16	2.7
Professors utilize the RUM principles in their practice	10.7	52	28	1.3	1.3
I have used the Brazilian Essential Medicines List for selecting medicines	8	10.7	22.7	57.3	1.3
The prescription of medicines in our hospital is influenced by information given by the pharmaceutical industries	4	37.3	29.3	16	10.7
I use to accept gifts from the pharmaceutical industries	32	20	13.3	28	6.6
It is easy to discuss a rational prescription in our hospital	6.7	40	41.3	4	8
I feel the conflict between what I learned on RUM and the medical practice in our hospital	20	60	9.3	9.3	1.3

A, always; ST, sometimes; R, rarely; N, never; IDR, I don´t remember.

Although 54.7% of the past students say they follow the RUM principles, 57.3% do not use the Essential Medicines List, which is intrinsic to RUM. The conflict between their knowledge and their practice has been perceived always by 20% of them and sometimes by 60%. Finally, 41.3% reported that the discussion of a rational prescription is not frequent, and encounters barriers within the Institution.

For the content analysis, the sample consisted of 12 past students. The in-depth interpretation of these data showed that:

1. former students had changed their perception on prescribing and became aware of the importance of a good prescription:

2. Former students seek information on medicines in text-books, scientific articles, but also in commercial sources:

3. Past students reinforce that they select medicines following the RUM principles and look for the best convenience and accessibility in free public health facilities for the patients:

4. Former students frequently comply with teachers' and senior residents' prescriptions in the University Hospital environment.

5. Former students suggest that hospital physicians should be capacitated in the RUM and this issue should be taught continually throughout the graduation course:

## Discussion

We verified the use of the RUM principles by medical students and residents who have attended the curricular discipline on this subject. This discipline, an unique experience in Brazil, is based on the WHO’s Guide to Good Prescribing [13]. This work was carried out in three steps: 1) elaboration and development of the discipline since 2003, 2) in 2010, we verified, through a questionnaire, if this type of knowledge was assimilated by our past students in their medical practice; 3) still in 2010, we performed a content analysis to deepen the evaluation of this assimilation.

The results of the assessment by students at the end of the discipline have allowed us to assume that the discipline is useful to teach RUM in a medical school. Our students were encouraged to think about the different variables that influence the selection of medicines and learned a logic deductive thinking they can always use to make their decisions. Additionally, they learned how to write a complete and accurate prescription.

However, the in-deep evaluation showed that although the majority of our former students recognize the importance of RUM, most of them have not been able to apply regularly this knowledge in their daily medical practice. They say they are not stimulated or driven to use of RUM because many senior residents and professors do not emphasize the principles of RUM, especially regarding to the written prescription. This finding suggests that the knowledge about RUM seems to interfere very little on the medical practice in our hospital.

RUM is a significant issue and in spite the fact that physicians are the major prescribers worldwide, its teaching is not a frequent practice in medical schools. This may be one of the major causes of prescription errors and the human and economic consequences that have been reported in the literature [2-4,14,15,21-26].

Currently there is a strong pressure for indiscriminate drug prescription. The society perceives drugs as a miraculous symbolic value, the prescribers feel obliged to prescribe drugs and are not adequately trained to do so, and the pharmaceutical industry aggressively promotes its new products, especially those that are not very innovating [27,28].

Therefore, the teaching of RUM which foresees a systematic approach for the selection of drugs and good prescription practices has been considered essential to correct or at least reduce these problems [14,22,29,30]. There are reports of different interventions for the teaching of several elements of RUM in medical graduation and specialization courses [30-36]. Results showed that very short interventions do not provide good results [37].

A systematic review by Ross and Loke [38] showed that there is no strong evidence whether educational interventions can improve prescribing by medical students and junior doctors; the selected studies reporting prescribing teaching for medical students or new doctors had small samples, were performed only at a single centre and/or presented different outcome measures. In the absence of evidence to support other interventions, the WHO model seems to be a good foundation for the design of a target prescribing curriculum, since it has been the only model widely used and shown some beneficial effects [36,38-41].

Unfortunately, until now there are not yet valid and reliable mechanisms for assessing written prescriptions and to evaluate the impact of this intervention on the health system.

The present work, which intends to bring a contribution to this subject, shows that despite the fact that the discipline is consistent and interesting, by itself it did not change very much the scenario of irrationality in the use of medicines. This situation requires, as recommended by the WHO [2,15], a continuous medical education program for training the physicians who work in the medical services in RUM so they can also develop these capabilities and competences. The involvement of the Institution seems to be fundamental in this process, creating a network among physicians, pharmacists, nurses, and the community to promote RUM.

## Conclusion

In conclusion, from our point of view this discipline is useful to teach RUM to medical students, but the assimilation of the RUM principles by the hospital medical staff seems to be a long-term process which requires the involvement of a greater number of the academic members.

## Competing interests

The authors declare that they have no competing interests.

## Authors' contributions

KPP conceived of the study, participated in its design, interpreted the content analysis, and drafted the manuscript. NABA carried out the qualitative approach and helped to draft the manuscript. NGA carried out the qualitative approach and helped to draft the manuscript. TTQ conceived of the study, participated in its design and coordination and draft the manuscript. All authors read and approved the final manuscript.

## Authors' information

^1^Assistant Professor, Department of Public Health, MD, PhD; ^2^Medical Residents; Department of Internal Medicine, MD; ^3^Full Professor, Department of Internal Medicine, MD, PhD.

## Pre-publication history

The pre-publication history for this paper can be accessed here:

http://www.biomedcentral.com/1472-6920/12/56/prepub
